# Trading water: virtual water flows through interstate cereal trade in India

**DOI:** 10.1088/1748-9326/abc37a

**Published:** 2020-12-01

**Authors:** Francesca Harris, Carole Dalin, Soledad Cuevas, Lakshmikantha N R, Tapan Adhya, Edward J M Joy, Pauline F D Scheelbeek, Benjamin Kayatz, Owen Nicholas, Bhavani Shankar, Alan D Dangour, Rosemary Green

**Affiliations:** 1 Department of Population Health, London School of Hygiene & Tropical Medicine, Keppel Street, London, United Kingdom; 2 Centre on Climate Change and Planetary Health, London School of Hygiene & Tropical Medicine, Keppel Street, London, United Kingdom; 3 Institute for Sustainable Resources, Bartlett School of Environment, Energy and Resources, University College London, London, United Kingdom; 4 Centre for Development, Environment and Policy, School of Oriental & African Studies, London, United Kingdom; 5 Ashoka Trust for Research in Ecology and the Environment, Bengaluru, India; 6 Manipal Academy of Higher Education, Manipal, India; 7 Kalinga Institute of Industrial Technology (Deemed University), Bhubaneswar, India; 8 Helmholtz Centre Potsdam German Research Centre for Geosciences, Potsdam, Germany; 9 Brandenburg University of Technology Cottbus-Senftenberg, Cottbus, Germany; 10 Department of Statistical Science, University College London, London, United Kingdom; 11 Institute for Sustainable Food & Department of Geography, University of Sheffield, Sheffield, United Kingdom; Francesca.Harris@lshtm.ac.uk

**Keywords:** food trade, agriculture, water resources, water savings, food security, food system, surface water

## Abstract

Cereals are an important component of the Indian diet, providing 47% of the daily dietary energy intake. Dwindling groundwater reserves in India especially in major cereal-growing regions are an increasing challenge to national food supply. An improved understanding of interstate cereal trade can help to identify potential risks to national food security. Here, we quantify the trade between Indian states of five major cereals and the associated trade in virtual (or embedded) water. To do this, we modelled interstate trade of cereals using Indian government data on supply and demand; calculated virtual water use of domestic cereal production using state- and product-specific water footprints and state-level data on irrigation source; and incorporated virtual water used in the production of internationally-imported cereals using country-specific water footprints. We estimate that 40% (94 million tonnes) of total cereal food supply was traded between Indian states in 2011–12, corresponding to a trade of 54.0 km^3^ of embedded blue water, and 99.4 km^3^ of embedded green water. Of the cereals traded within India, 41% were produced in states with over-exploited groundwater reserves (defined according to the Central Ground Water Board) and a further 21% in states with critically depleting groundwater reserves. Our analysis indicates a high dependency of Indian cereal consumption on production in states with stressed groundwater reserves. Substantial changes in agricultural practices and land use may be required to secure future production, trade and availability of cereals in India. Diversifying production systems could increase the resilience of India’s food system.

## 1. Introduction

Rising global population and economic growth are increasing pressure on global water resources [1]. An estimated four billion people experience severe water scarcity for at least one month of the year, where the water demand exceeds that available for use locally [2]. The agricultural sector dominates human water use and is particularly vulnerable to water scarcity [3, 4]. Currently, 20% of global irrigation is dependent on groundwater abstraction from depleting aquifers [5], and the greater frequency of extreme weather events is threatening agricultural productivity [6, 7]. Understanding the trade of food, and its embedded or virtual water, can illustrate linkages between food consumers and water resources, and identify when changes in the availability of water might affect the availability of food [8–11].

Indian agriculture plays a major role in national and global food security, and is a source of employment for over half of the Indian workforce [12]. Indian population growth is leading to increased demand for food and water [13]. Greater use of improved crop varieties, irrigation and fertilisers have contributed to substantial improvements in crop yields in India [14]. However, in the major food-producing states of Punjab, Haryana and Uttar Pradesh in the north, and Tamil Nadu and Karnataka in the south, groundwater resources are rapidly depleting [15]. Recent shifts to greater food production in the dry season to avoid unreliable rainfall in the wet season may further increase dependency on ground- and surface-water for agricultural irrigation [16]. Cereals are an important component of the Indian food system, comprising 45% of all agricultural production [17], and contributing to 47% of the total daily dietary energy intake. India is self-sufficient in cereals [18], importing only 0.01% of national cereal supply from other countries, and is a major exporter of rice and wheat globally [19].

This study aims to quantify the interstate and international trade of cereals in India, and the associated trade of embedded or virtual water. We extend previous estimates of virtual water trade in India that have either focused only on international trade [20, 21]; estimated the embedded water in food grains transported by railways [22] (20% of all food grain transport [23]); focused only on trade through the Indian Public Distribution System (PDS—a large-scale Government programme that procures and redistributes cereals at fair priced shops) that contributes to 35% of all cereal consumption [24]; or have not accounted for the PDS [25]. Our study explores the totality of the virtual water trade associated with cereals in India by developing a model to predict interstate cereal trade flows through both road and rail transport, and fully incorporating both the PDS and international trade. The primary objective of this study is to enhance understanding of the dependency of the Indian food system on water resources.

## 2. Methods

### 2.1. Estimating supply and demand of cereals in each state

There are currently no comprehensive data available on interstate cereal trade in India (hereafter, ‘state’ refers to State and Union Territories [*N* = 35]). We quantify the trade of cereals through the PDS and non-PDS cereals separately.

For each of the five major cereals consumed in India (wheat, rice, maize, millet and sorghum; 99% of total cereals available for human consumption [19]), state-level data were collated on production, foreign imports and exports, PDS procurement, stocks, non-food uses and amounts available for food consumption. The supply by states of each cereal includes local production and foreign import, plus net change in stock (i.e. cereals stored between production and retail), waste and non-food uses of cereals (feed, seed, processed and other). The demand by states for each cereal includes food consumption and foreign export. We estimated interstate cereal trade by modelling non-PDS cereal supply and demand balance, where excess supply from a state meets unmet demand in other states, and used data on PDS procurement and consumption to estimate PDS trade.

Analysis was focused on the years 2011–12 as this is the most recent time period for which all required data were available. Data were collated from various sources as follows (full details in supplemental table S3, which is available online at https://stacks.iop.org/ERL/15/125005/mmedia): state cereal production was derived from Government production statistics; the proportion of cereal supply wasted and allocated to non-food uses were taken from India-specific data from the Food and Agricultural Organisation (FAO) Food Balance Sheets [19]; and data on PDS procurement were used to estimate the amount of rice and wheat exported through the PDS [26]. The total volume of foreign imports and exports for India was estimated following methods from Kastner *et al*, whereby global data on bilateral trade flows is integrated with country-level production estimates to account for the origin of production and final destination of commodities rather than representing port stops [27]. There are no data available on production by states for international export or on the consumption of foreign cereals. Therefore, to link international trade with domestic trade we used data from the Agricultural & Processed Food Products Export Development Authority, Ministry of Commerce & Industry [28] that specifies port of entry and exit for commodities. Total volume amounts of foreign imports and exports estimated following methods of Kastner *et al* [27] were allocated proportionally to port states (*N* = 13) based on these port import and export quantities. Foreign imports were integrated into the port state’s supply of cereals, and foreign exports were to the port state’s demand along with food consumption. The quantity of cereals required for food consumption was estimated from the 68th Round of the Indian National Sample Survey (NSS) conducted in 2011–12 [24]. The NSS is a nationally representative household consumption and expenditure survey conducted by the Government of India, that does not include food eaten outside the home and therefore underestimates consumption. Hence, we calculated consumption using the availability of each cereal after removal of non-food uses and foreign export from the supply in each state, and estimated the consumption by dividing the total availability for food at national level by the proportional consumption for each state according to NSS.

The supply and demand accounts were used to identify states with excess cereals for interstate trade and states with unmet demand. For each cereal, states with supply greater than their own demand were designated as cereal exporters, while states with demand greater than their supply were designated as importers.

### 2.2. Quantifying domestic and foreign cereal trade in India

The direction and volume of non-PDS cereal trade flows were estimated through a linear programming model that minimised the overall cost of transportation [23, 29–31]. Previous analysis on intra-national trade flows suggests that models that minimise the cost of transport provide estimates are comparable to primary data [32]. The methods are briefly described below (see supplemental file for equations and a full list of data sources).

The cost of transportation between states was calculated based on the rail and road distance to each respective state capital, multiplied by the cost of transportation per km per tonne of cereals for each mode. Minimum road distance was estimated using map data from Google [33], and minimum rail distance for commodity transport was taken from the Indian Government Centre for Railway Information Systems online tool [34]. Using data from the Indian Government Planning Commission on the cost of transportation per km per tonne of cereals (as the food group) [35], we calculated the associated transportation cost matrix for each mode. The relationship between transportation cost and distance travelled is non-linear, as it is assumed longer routes will have reduced time and capacity costs relative to shorter distances. The transportation cost to and from the island states (Lakshadweep and Andaman and Nicobar Islands) includes the cost of shipment to their nearest mainland ports according to the shipping distance and cost per km per tonne for shipment [35], and the cost of rail or road transport between the state of their mainland port and other states. A combined cost of transportation matrix for cereals between Indian states was estimated using the proportion of cereals (as the food group) transported by road or rail in India [35], and subsequently used as the cost to be minimised in the linear programming model.

An optimisation model was constructed with the objective function to minimise transportation costs, while allocating the excess supply from states to those with unmet demand for each cereal. The constraints for the model were as follows:

• Supply of each commodity equals demand in each state.
• Trade flows are only positive.
• Foreign imports are added to the port states’ total supply, while foreign exports are added to the port states’ demand.
• Net export of the commodity is bounded by local production or foreign import (if any).

The model was run independently for each cereal, giving an output of total tonnes of each cereal traded annually between every combination of two states.

To validate the approach of minimising transportation costs to estimate non-PDS trade, we used a mixed effects linear regression model to assess the association of our calculated cost of transportation (Rupees kg^−1^) for importing each cereal with the value of the corresponding cereal to the consumer in the importing state (Rupees kg^−1^ — using data from the NSS). The cost of transportation was weighted according to import volume from each exporting states, as calculated by the trade model.

We considered separately the trade of cereals through the government PDS programme that procures rice, wheat and other crops at a minimum support price and sells these at a reduced rate in fair price shops. The PDS does not distribute based on minimising the cost of transportation [36, 37], hence we did not use the optimisation model to estimate PDS trade. Data is available on the volume of rice and wheat procured by the central Indian government for the PDS [26]. We calculated the volume of PDS exports for each state based on the known contribution to the central pool after the removal of waste (according to national average proportions). We assumed that states import PDS cereals from this central pool proportionally to their estimated PDS consumption in the NSS [24]. For states with a decentralised PDS (*N* = 13) (i.e. they satisfy their own PDS demand, but still contribute to central pool), PDS consumption was calculated according to proportional PDS rice and wheat consumption compared to non-PDS rice and wheat consumption in the NSS. Total PDS production in India reflects the total procurement of PDS cereals and the estimated local PDS consumption in decentralised states.

We evaluated the association of common drivers of trade (e.g. distance, GDP) with interstate trade patterns for non-PDS and PDS cereals through a gravity model (see supplemental file section 1.4 for full details on the gravity model methods). We compared whether our model outputs on estimated trade flows were consistent with existing gravity models of interstate trade flows on the rail trade of agricultural commodities [38], and the trade of manufacturing goods [39].

Data matching and cleaning was carried out in MS Excel and R Studio (R Version 3.6.1). The linear programming model was run in R Studio using integer programming for solving transportation problems (available through the lpSolve package in R, see supplemental file section 1.2.2 for code) [40]. Interstate trade matrices for each cereal are avaialble at Harris *et al* [41].

### 2.3. Quantifying virtual water trade

State-level blue and green water footprints (WFs) were used to calculate virtual water trade (see supplemental file section 1.5 for detailed equations). The green WF refers to the volume (m^3^/tonne) of precipitation water that is consumed during crop production, either from evapotranspiration, transpiration, or incorporated into the final crop product [42]. The blue WF refers to the volume (m^3^/tonne) of water withdrawn from ground- and surface-water sources and consumed during crop production, or incorporated into the final crop product [42]. The state-level WFs of domestic cereal production were taken from published data covering the years 2005–14 [16] that were estimated using an online WF assessment tool [43] and government production and irrigation statistics. These WF estimates are slightly lower than published data from earlier years (1996–2005) [42], due to improved yields and a small decrease in reference evapotranspiration. Full methods and comparison to other WFs can be found in Kayatz *et al* [43].

The WFs of foreign imports were weighted according to import volume from each country of origin. WF values of foreign cereals were only available from the years 1996–2005 [42], however foreign imports contribute very little (<0.01%) to the total supply so this will not substantially affect our virtual water trade estimates. Virtual water trade was calculated as the product of cereal export and associated cereal WF in the exporting state (in m^3^/tonne). For port states exporting both domestic and foreign cereals, the WFs of cereal exports were weighted based on the amount of domestic and foreign cereals in the port state’s supply.

We further explored the ground- and surface- virtual water trade of domestically produced cereals. State-level blue WFs were proportionally weighted according to state-level data on the area irrigated by ground- and surface-water [44, 45]. Ground- and surface-water trade was only estimated for domestically produced cereals as the required data were not available for foreign imports. We matched cereal exports to the groundwater status of the exporting state in 2011–12 as defined by the Central Ground Water Board [46] that categorised states as safe, semi-critical, critical and over-exploited according to ratio of groundwater use to groundwater availability [47]. To illustrate interstate trade patterns we constructed chord diagrams using the ‘circlize package’ in R that displays trade pairs in a circle format using chords that are proportionally sized to the volume of trade between trading pairs [48].

Finally, we calculated theoretical green, ground- and surface-water savings due to interstate cereal trade. A trade relationship is considered to lead to water savings when crops are exported from a relatively more water-productive state (i.e. where the crop has a lower WF) to a less water-productive state [29]. Trade flows in the opposite direction are considered to lead to negative water savings, i.e. water losses. In other words, water savings represent the difference between water that would be used to produce cereals for food consumption in a no-trade situation and the water currently used. The practical meaning of this needs to be carefully considered as the quantity of the crop imported by a state cannot always be produced locally. Water savings were calculated for each cereal and each trading pair of states. National water savings represent the sum of savings for all the interstate trade links.

### 2.4. Sensitivity analysis

We explored the sensitivity of our model and virtual water trade estimates to the input data. To illustrate the sensitivity to assumptions on cereal transport modes and costs, we estimated the trade patterns of non-PDS cereals that would occur if transport between states was conducted only by rail or only by road. We also carried out a sensitivity analysis to explore the assumption that the PDS does not trade cereals across states in a way that would minimise the cost of transportation. We used a linear programming model that minimised the cost of transportation that would be required to balance supply and demand of PDS rice and wheat across Indian states (as for non-PDS cereals). Finally, sensitivity analysis was carried out using annual average production, foreign trade quantities and allocation of cereals to non-food uses for the years 2010 to 2013. We compared these trade patterns and results with the 2011–12 model, in order to test the robustness of our conclusions to annual fluctuations in cereal supply.

## 3. Results

### 3.1. Overview of cereal production, consumption and foreign trade

We first present for the study period (2011–2012) an overview of cereal production, consumption, and foreign trade and the associated embedded water. The annual cereal production in India for 2011–12 was 249.9 million tonnes (Mt), of which 42% was rice, 41% wheat, 8% maize, 6% millet and 3% sorghum (table 1) [17]. The volume of embedded water in these cereals amounted to 292.3 km^3^ of green water, and 145.3 km^3^ of blue water. After accounting for the non-food uses of cereals (feed, seed, processing), waste, and foreign export, 201.2 Mt of cereals remained in India for human food consumption (81% of total production). The embedded water use of cereal consumption was estimated as 237.3 km^3^ of green water and 123.9 km^3^ of blue water. Foreign imports made a very small contribution to total cereal supply and nearly 10 Mt of cereals (with an associated 14.4 km^3^ of embedded water) were exported.

**Table 1.** Estimated production, consumption and foreign trade of cereals in India and the associated embedded water, for the period 2011–12. PDS: Public Distribution System.

Variable	Total volume (Mt)	Embedded green water (km^3^)	Embedded blue water (km^3^)
Total cereal production	249.9	292.3	145.3
Cereal production for the PDS	74.2	71.3	48.5
Total cereal allocated to food consumption	201.2	237.2	123.9
Cereal consumption through the PDS	71.4	68.6	46.7
Foreign import	<0.1	<0.1	<0.01
Foreign export	9.7	9.9	4.5

### 3.2. Interstate trade of cereals and the associated virtual water trade

**Figure 1.**
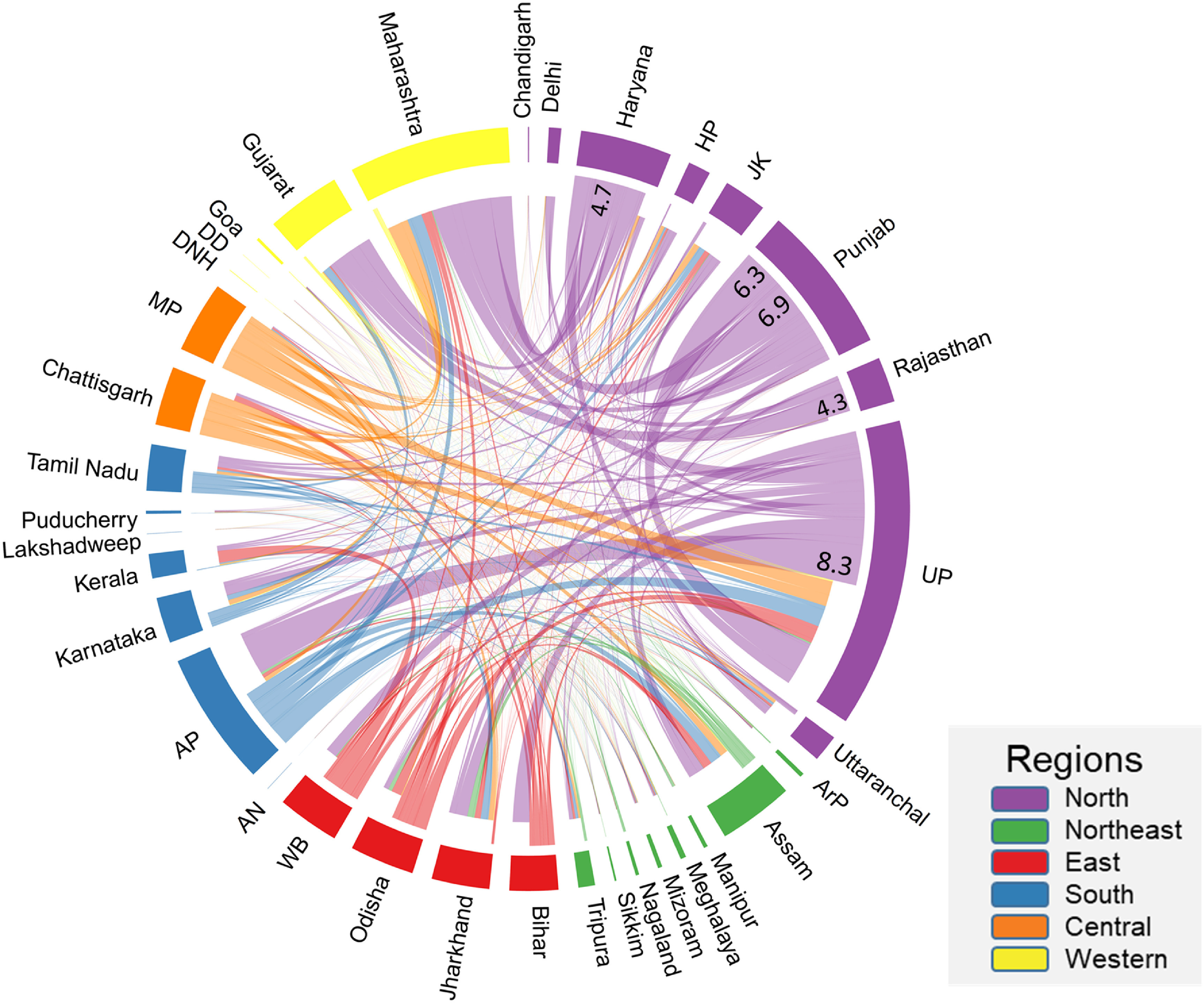
Total virtual water embedded in interstate trade of cereals in India during 2011–12, in cubic kilometres per year. Chords link exporting and importing states, and are coloured according to region of the exporting state (see supplemental file figure S1 for map of Indian states and regions). Chords are indented for importing state. Some states are abbreviated for figure clarity: HP: Himachal Pradesh, JK: Jammu and Kashmir, UP: Uttar Pradesh, ArP: Arunachal Pradesh, WB: West Bengal, AN: Andaman and Nicobar, AP: Andhra Pradesh, MP: Madhya Pradesh, DNH: Dadra and Nagar Haveli, DD: Daman and Diu.

We estimated that 93.8 Mt of domestic- and foreign-produced cereals were traded for food consumption between Indian states during 2011–12 (40% of the total food supply of cereals in India). The main cereal traded was rice (45.5 Mt, 48% of cereal trade), followed by wheat (40.0 Mt, 43% of cereal trade). The total water embedded in interstate cereal trade was equal to 153.4 km^3^ (figure 1), of which 35% (54.0 km^3^) was blue water, and 65% (99.4 km^3^) was green water (see supplemental files, figure S3 for trade patterns in Mt and virtual water trade flows separated out by PDS and non-PDS trade, and type of water).

There were regional and state-level differences in the contribution to interstate imports and exports. The Northern region accounted for 61% of all cereal interstate exports (56.9 Mt), equivalent to 83.8 km^3^ of embedded water. The Western region exported the least amount of cereals: 1.0 Mt (1%), equivalent to 1.7 km^3^ of water. There were 5 states that imported but did not export cereals to other states: Chandigarh, Delhi, Lakshadweep, Manipur, and Mizoram. States that imported the largest amount of water through cereal trade were Maharashtra (28.4 km^3^; 11.5 Mt), and Uttar Pradesh (24.8 km^3^; 7.1 Mt).

Trade patterns varied between PDS and non-PDS cereals. The majority (58%; 58.0 Mt) of interstate cereal trade occurred through the PDS. The total volume of embedded water traded through PDS rice and wheat amounted to 54.3 km^3^ of green water and 36.7 km^3^ of blue water. As the main PDS contributors, the states exporting the most water through the PDS were Punjab (20.9 km^3^), Andhra Pradesh (12.6 km^3^), and Madhya Pradesh (9.9 km^3^).

In addition, 35.8 Mt of non-PDS cereals were traded between states, corresponding to 45.1 km^3^ of green water and 17.3 km^3^ of blue water. The Northern region accounted for 78% of these blue water exports and 67% of the green water exports.

### 3.3. Virtual water trade of domestically produced cereals according to groundwater status in the exporting state

**Figure 2.**
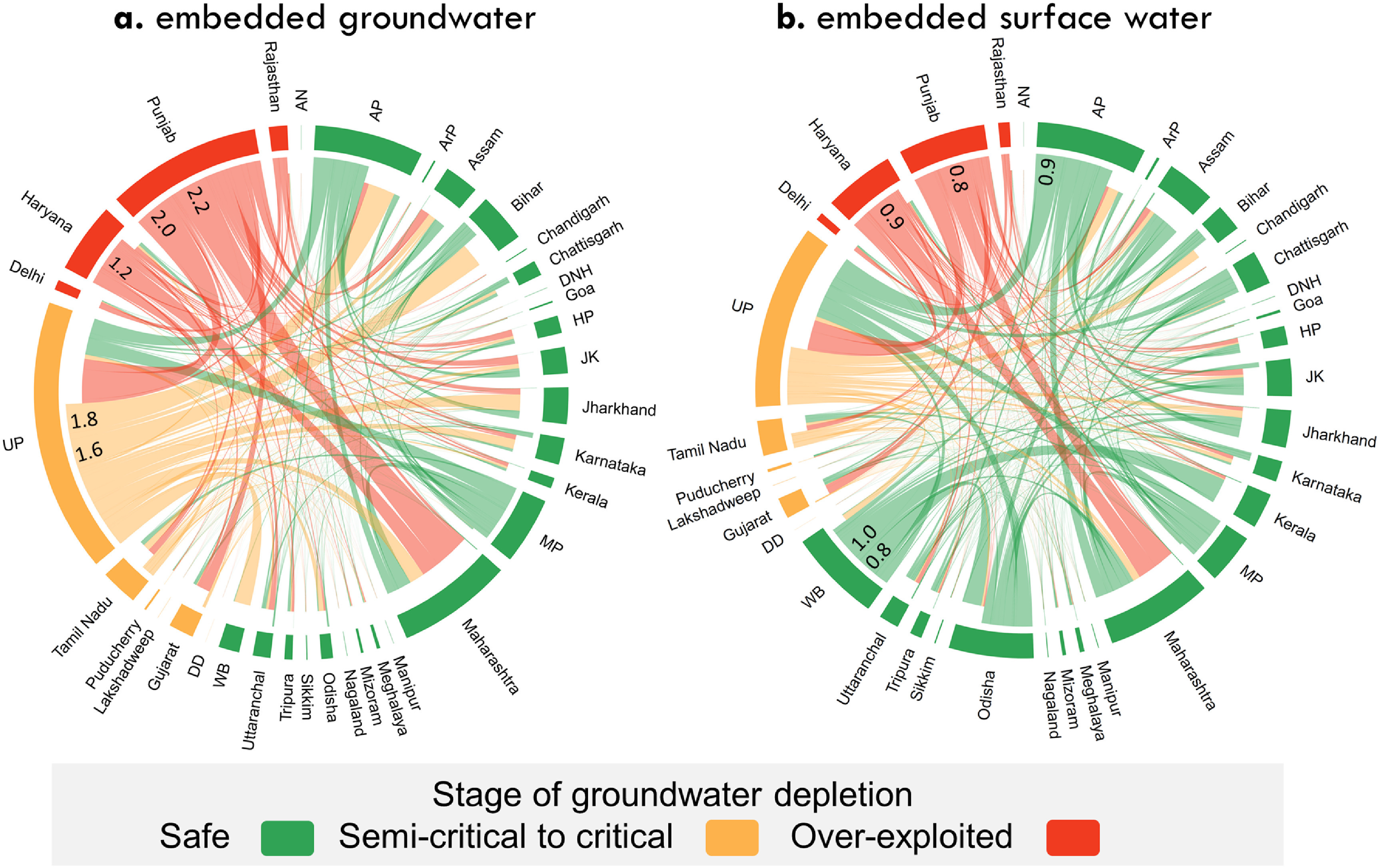
The ground- and surface-water embedded in the interstate trade of cereals in India during 2011–12, in cubic kilometres per year. Chord colour corresponds to the stage of groundwater depletion in the exporting state in 2011, as defined by the Central Groundwater Board of India [46]. Chords are indented for importing state. Some states are abbreviated for figure clarity. AN: Andaman and Nicobar, AP: Andhra Pradesh, ArP: Arunachal Pradesh, DNH: Dadra and Nagar Haveli, HP: Himachal Pradesh, JK: Jammu and Kashmir, MP: Madhya Pradesh, DD: Daman and Diu, UP: Uttar Pradesh, WB: West Bengal.

We explored the patterns of trade and the embedded ground- and surface-water of domestically produced cereals according to status of groundwater depletion in the exporting state. Nearly all (99.9%) of the cereals traded between Indian states were produced domestically. The embedded water in interstate trade of domestically produced cereals was equal to 32.3 km^3^ of groundwater and 21.7 km^3^ of surface water (table 2, figure 2, see supplemental file figure S4 for results separated out by PDS/non-PDS trade).

**Table 2.** Trade of domestically produced cereals and the embedded surface water and groundwater, according to groundwater status in the exporting or importing state. Groundwater status defined according to the Central Groundwater Board estimates from 2011. PDS: Public Distribution System. Row percentages may not total 100% due to rounding.

Variable	Status of groundwater in states		
	Safe (*N* = 25)	Semi-critical to critical (*N* = 6)	Over-exploited (*N* = 4)
**Total state exports of domestically produced cereals (Mt, % of row total)**	35.5 (38%)	19.6 (21%)	38.6 (41%)
PDS exports (Mt, % of row total)	24.4 (42%)	6.4 (11%)	27.2 (47%)
Non-PDS cereal exports (Mt, % of row total)	11.2 (31%)	13.2 (37%)	11.4 (32%)
**Embedded groundwater in state exports of domestically produced cereals (km^3^, % of row total)**	9.2 (28%)	10.4 (32%)	12.7 (39%)
**Embedded surface water in state exports of domestically produced cereals (km^3^, % of row total)**	12.7 (59%)	3.1 (14%)	5.9 (27%)
**Total state imports of domestically produced cereals (Mt, % of row total)**	63.8 (68%)	26.2 (28%)	3.8 (4%)
PDS imports (Mt, % of row total)	36.6 (63%)	19.5 (34%)	2.0 (3%)
Non-PDS cereal imports (Mt, % of row total)	27.2 (76%)	6.7 (19%)	1.8 (5%)
**Embedded groundwater in state imports of domestically produced cereals (km^3^, % of row total)**	22.7 (70%)	8.2 (25%)	1.4 (4%)
**Embedded surface water in state imports of domestically produced cereals (km^3^, % of row total)**	15.1 (70%)	5.9 (27%)	0.7 (3%)

States defined as over-exploited in their groundwater reserves according to the Central Groundwater Water Board of the Government of India [46] (*N* = 4) produced and exported 41% (38.6 Mt) of the domestically produced cereals in interstate trade (table 2), equivalent to 39% (12.7 km^3^) of the total groundwater embedded in interstate cereal trade. A further 21% (19.6 Mt) of domestically produced cereals were exported from states with semi-critical to critical groundwater status (*N* = 6), equivalent to 10.4 km^3^ (32%) of groundwater. States with over-exploited groundwater resources imported 4% of cereals (3.8 Mt), equivalent to 1.4 km^3^ of groundwater.

States with safe groundwater reserves (*N* = 25) exported 35.5 Mt (38%) of domestically produced cereals, equivalent to 9.2 km^3^ (28%) of the embedded groundwater traded between states, and imported 63.8 Mt (68%) of cereals, equivalent to 22.7 km^3^ (70%) of groundwater. These states were the main contributors to virtual surface water exports through domestically produced cereals (12.7 km^3^; 59%).

PDS trade was more dependent on over-exploited groundwater than non-PDS cereal trade; 47% of PDS cereal exports (27.2 Mt) came from states with over-exploited groundwater resources compared to 32% of non-PDS cereal exports (11.4 Mt). States with groundwater resources defined as safe imported 63% of PDS cereals (36.6 Mt) and 76% of non-PDS cereals (27.2 Mt).

### 3.4. Water savings induced through trade

Trade-induced water savings in India during 2011–12 amounted to 28.8 km^3^ of green water and 4.5 km^3^ of surface water. However, there was a theoretical loss of groundwater resources due to trade of 2.0 km^3^. For 27 states, cereal trade was groundwater-inefficient, i.e. these states had a lower groundwater WF per tonne than the states from which they imported (see supplemental figure S5 for water saving by state).

### 3.5. Validation of the model and sensitivity analysis results

#### 3.5.1. Validation of the cost of transportation data

We found strong evidence that the cost of transportation as estimated in this study was associated with the unit value paid by the consumer, validating our use of a minimal cost optimisation model for this analysis (see supplemental file figure S6 for scatter plot). For every 1 Rupee kg^−1^ increase in the cost of transportation, the price of cereal for the consumer increased by 4.92 Rupees kg^−1^ (95% CI 1.58 to 8.06, *P* < 0.01, *N* = 114).

#### 3.5.2. Comparison of the trade model to existing literature

We used a gravity model to compare the modelled cereal trade flows with existing data on rail trade flows of agricultural products from 2005–14 [38], and the trade of manufacturing goods from 2015–16 [39]. The gravity model analysis of non-PDS cereals demonstrated that non-PDS cereal trade was primarily driven by distance; consistent with existing evidence on international trade flows [49] and interstate trade flows of manufacturing goods in India [39]. The gravity model including PDS and non-PDS cereal trade identified that distance was not a barrier to trade; consistent with the fact that PDS does not distribute cereals based on minimising the cost of transportation, and in line with existing evidence on agricultural rail trade in India that included PDS cereals [38]. The good alignment of our findings from gravity models with the existing evidence base supports the validity of our approach to model interstate cereal trade in India and also suggests that our results are reflective of interstate trade patterns from a broader time frame (see supplemental file, section 2.4 for full results).

#### 3.5.3. Sensitivity analysis using varying cost of transportation

We explored the sensitivity of the estimated non-PDS cereal trade flows to the cost of transportation data by comparing our modelled results to cereal trade conducted only by rail or by road. Trade patterns were similar under each mode, with the Northern region again dominating cereal exports (supplemental file figure S7). Compared to the combined transport mode model, the volumes of cereal traded varied for 114 (11%) trading pairs under road transport and 131 (19%) trading pairs under rail transport. This equates to 4.0 Mt (11%) and 14.9 Mt (41%) of cereals traded differently under road transport and rail transport respectively, identifying that our modelled trade flows were sensitive to assumptions on mode of transport.

#### 3.5.4. Sensitivity analysis of cereal trade through the Public Distribution System

We modelled PDS trade based on minimising the cost of transportation to compare with our main results. We found that interstate trade of PDS cereals was reduced by 9% to 53 Mt, as more cereals remained in the state where they were produced to minimise the cost of transport (see supplemental file figure S8 and table S5). Additionally, there was a slight shift in the proportion of cereals exported from states according to groundwater status: 52% (27.6 Mt) of cereals were exported from states defined as over-exploited in their groundwater reserves, compared to 47% (27.2 Mt) in the central pool model.

#### 3.5.5. Sensitivity analysis using supply data from a different time frame

To test the sensitivity of our findings to the input data on cereal supply (production, international trade and stock), we quantified the virtual water trade network using yearly average production, stock and international trade data over the period 2010–13. Trade patterns obtained from the 2010–13 data were similar to the 2011–12 estimates, such that the Northern region dominated exports and the Western region imported the most (see supplemental file, section 2.3 figures S9 and S10 for chord diagram using 2010–13 data, and table S6 for comparison of key variables using 2010–13 and 2011–12 data). The largest differences in regional trade patterns of non-PDS cereals were imports in the Northeast region, and exports from the Western region. Imports in the Northeast were greater using 2010–13 average at 2.8 Mt compared to 0.6 Mt in 2011–12, and the Western exports were greater using 2010–13 average at 1.6 Mt compared to 0.5 Mt in 2011–12.

The total volume of virtual water traded between Indian states through cereals was estimated to be 152 km^3^ using the 2010–13 average, which is 1.0% lower than our estimate from 2011–12. The estimated volumes of ground-and surface-water embedded in the trade of domestically produced cereals were 32.5 km^3^ and 22.1 km^3^ respectively, which are marginally larger (by 0.8% and 1.8%, respectively) than the values calculated for 2011–12. As trade patterns varied slightly using the 2010–2013 average, so did the theoretical water savings induced by trade at national level. However, water savings followed the same pattern, such that green water savings were the greatest (35.4 km^3^ year^−1^), followed by surface water savings (1.92 km^3^ year^−1^), and there was a loss of groundwater resources (−3.18 km^3^ year^−1^).

## 4. Discussion

### 4.1. Summary

We built a supply and demand balance model that minimised transportation cost and combined with existing data to explore interstate trade of cereals for human food consumption and the associated virtual water flows in India. We estimate that 93.8 Mt of domestic- and foreign-produced cereals were traded for food consumption between states in 2011–12 with an associated total virtual water flow of 153.4 km^3^. States with over-exploited groundwater (as defined by the Central Groundwater Water Board of the Government of India) produced 41% of the interstate exports of cereals, and a further 21% was produced and exported from states with semi-critical or critical groundwater reserves. Through the interstate trade of cereals, 31 out of 35 Indian states rely at least in part on cereals produced in states with over-exploited groundwater, equating to 917 million people, or 76% of the Indian population. Our analysis of trade-induced water savings demonstrates that Indian interstate trade encourages the production of crops that use less rainwater and surface water in their production, but leads to slightly more groundwater use per year: 2.0 km^3^, equivalent to 2% of the total groundwater used for cereal production. Changes in production and interstate trade patterns, in irrigation methods and in the type of cereal consumed appear necessary to improve the resilience of India’s food system.

### 4.2. Research in context

There are many studies that explore the impact of food consumption on water use at the national level [50]. However, water requirements vary between crops, and are affected by local agricultural and climatic factors; hence in a country the size of India, estimating the embedded water in food consumption and assessing the associated resilience of the food supply, requires subnational information linking locations of consumption and production. The blue water use of cereal consumption in 2011–12 varies by more than 1000% for some states if local WFs are used rather than trade-weighted WFs (supplemental file, table S7), demonstrating the value of understanding patterns of within-country trade when assessing the environmental impacts of food systems [9].

Our findings have particular relevance for Indian water management policies that aim to address the unequal distribution of water resources. The National River Interlinking project, a major infrastructure scheme supported by the Indian Government, aims to transfer water from water-abundant to water-scarce regions. It has been estimated that once this project is completed a total of 175 km^3^ year^−1^ will be transferred from the Eastern region where groundwater reserves are not stressed, to the major food producing regions in the North [51]. Consistent with previous assessments, we show that virtual water currently moves in the opposite direction through trade of food crops, from north to east. Our estimate for the total water transferred through cereal trade is slightly less than the estimated water flow through canals and rivers in the interlinking project at 153 km^3^ year^−1^. This is higher than previous estimates, as we have accounted for both PDS and non-PDS cereal trade, and incorporated internationally imported cereals. Our findings reiterate the substantial potential for balancing water resources through the trade of crops in India, either in addition to or in place of large-scale infrastructure projects.

The patterns of interstate cereal trade in India emphasise the large dependency of agriculture on groundwater irrigation in groundwater-scarce states. Similar relationships have been found for intra-national trade in the United States of America [52]. Water policy is currently set at state level in India [53]. Our analysis suggests that a national-level perspective on water resource use is needed to understand supply risks and opportunities for effective integrated water resource management. Electricity subsidies for agriculture provided by state governments have encouraged farmers to extract groundwater at increasing depths [54, 55]. We found that the interstate trade of cereals is associated with slightly more groundwater use than there would be without such trade. It is possible that interstate cereal trade encourages continued production of cereals irrigated with groundwater for export. This may discourage agricultural improvements in importing states; Eastern states which are safe in their groundwater reserves and net importers, also have the highest yield gaps and therefore the greatest unmet potential to increase production [56, 57]. Adapting the agricultural subsidy system, for example by changing tariffs on electricity in the Eastern region [54], could help diversify cereal production locations in India, while interstate trade can be used to fulfil demand. Furthermore, diversifying the type of cereal produced could also reduce water use. Agricultural policies from the Green Revolution in India encouraged production of high-yielding rice and wheat and reduced emphasis on traditional cereal crops such as sorghum and millet [58]. Compared to rice and wheat these traditional cereal crops require less irrigation per tonne of production, are more drought resistant, and have greater nutritional quality. Therefore planting sorghum and millet in water scarce regions could reduce the total water used in Indian agriculture, improve resilience against future water shortages and lead to nutritional benefits [59, 60]. Other states could substitute some of the supply gaps in rice and wheat that can subsequently be traded to satisfy demand. Water availability is only one determinant of production diversity in India, and other factors including agro-ecological suitability, adaptability of production systems and infrastructure capacity, and the willingness of consumers to change consumption patterns of cereals should also be considered.

### 4.3. Limitations

Our study aimed to quantify interstate cereal trade in India and the associated virtual water trade. As with all modelled analyses, the results should be taken as representative of the likely reality. An important assumption of the trade model is that states will only export cereals if they have met their own consumption needs and, conversely, states will only import cereals if they have insufficient supply. This is a common assumption in supply and demand balance models, and has been used in previous sub-national trade analyses [32]. However, it has likely underestimated interstate trade. Additionally, we assumed that foreign products would be consumed by the port state before exporting to other states as international trade would be organised to limit the distance to markets in India, but this may not always be the case. Furthermore, we incorporated foreign exports as part of the port state’s demand, hence this must be imported from other states if it cannot be met by the port state’s supply. This would have accounted for some international trade occurring via ports in other states, but we may have underestimated the foreign export from certain states that have specialised production of higher quality cereals for export. Finally, the objective of the model was to minimise the cost of transportation, and because of the absence of data, transportation costs were necessarily estimated based on distance between state capitals as sites of the central cereal trade markets. While our model outputs suggested that adjacent states were more likely to trade than more distant states (supplemental file table S4), which is highly plausible, our approach will undoubtedly have affected estimates for transportation cost, particularly in larger states. Additionally, our transportation costs were estimated by the proportion of road and rail transport at national level, but this may vary for some states pairs. Our sensitivity analysis using just road or just rail transport indicated this assumption could affect trade flow estimates. Furthermore, the transportation costs were not disaggregated by cereal type, as data were only available for the cereal food group. Although transport logistics, such as storage, are likely to be similar across the cereal types, transportation costs or modes may vary due to differences in infrastructural capacity in the producing regions [61]. Despite these limitations, our cereal transportation cost estimates were found to be correlated to a higher unit value paid by consumers for the cereals. The large effect of transportation costs on unit value (4.92 Rupees kg^−1^ increase in price for 1 Rupee kg^−1^ in transportation cost), suggests the existence of additional costs along the supply chain, such as storage, intermediation or marketing costs. Additionally, cereal unit value differentials across Indian states are driven by difference in quality, as well transport costs [62]. It was not possible to disaggregate cereal trade by quality due to lack of data.

Using data on central procurement of cereals and estimates on PDS consumption from NSS data we proportionally allocated rice and wheat based on states’ demand and supply. In our model, states with an established decentralised system first satisfied their own PDS demand before exporting excess supply. It is possible that if all states do the same to minimise transportation costs the amount of interstate trade would decrease. Our sensitivity analysis exploring PDS trade suggests that minimising transportation costs would only reduce PDS trade by 9%, and mainly reduce exports from states with safe groundwater reserves. Therefore, while the assumption that PDS cereals are distributed from a central pool may overestimate trade pairs, it does not affect our conclusion that PDS trade is heavily dependent on exports from states with unsustainable groundwater use.

There are some limitations to the data. Our analysis has focused around a short time frame of 2011–12 as this was the most recent year for which all required data were available. While some factors that drive trade are relatively fixed including distance or agricultural land area for each state, other factors including rainfall patterns, cereals price and demand will vary over time. The quantity of cereals exported from some regions varied using 2010–13 yearly average supply estimates, which was possibly related to the droughts in 2010 that would have disrupted agricultural production in rainfall dependant states [63]. There were no major droughts or other extreme weather events in 2011–12 in India, hence this time period may be more reflective of normal trade patterns [13, 16, 63]. However, despite small differences in trade flows, the major trade pattern did not differ substantially between the two time periods and virtual water trade flows were comparable, supporting the robustness of our findings (supplemental file section 2.7, table S6). Nevertheless, the current and future status of Indian agriculture and water availability may be different to our study timeframe. Our estimated cereal consumption levels may not reflect recent years due to population growth and changes in cereal consumption patterns, but there are no recent data on cereal consumption at state-level that would allow us to explore this further. There have been no large changes to groundwater status in Indian states since the time period studied [64], but increased frequency of extreme weather events and changing precipitation patterns are altering agricultural practices [65, 66], which could affect water use. Continued monitoring of virtual water use and trade in India is warranted.

Our estimated total mass of cereals available for consumption at national level (Mt) was 9% and 29% higher than the equivalent values FAO’s Food Balance Sheets [19] and NSS [24], respectively (supplemental file table S1). Differences were lower for rice and wheat compared to other cereals. These discrepancies may be due to inaccurate estimates of the waste and non-food uses of cereals, for example it is possible that we underestimated leakage (waste) from the PDS, which may be up to 40% in some areas [67], hence we may have overestimated the consumption of PDS cereals. Neither NSS nor FAO’s Food Balance Sheets accurately assess total dietary consumption so discrepancies with the consumption values calculated in this study are expected. NSS underestimates food eaten outside the home and the consumption of processed foods, therefore it is possible that our estimates for state-level consumption may not accurately reflect the pattern of cereal consumption. However, the proportion of meals eaten outside the home does not vary appreciably across income levels or states [68], hence the consumption values estimated in our state are still reliable.

Finally, the objective of this study was to quantify the virtual water trade of cereals associated with human food consumption to illustrate relationships between food security and water resources, but cereals are only one (albeit the largest) food group. The virtual water trade of other crops, such as fruits, vegetables, and pulses, may be different. Additionally, cereals are also traded for feed for animal-sourced food, which was not included in our trade estimates. This will have underestimated the cereal trade, particularly for maize as 37% of production is used for feed in India according to India-specific data from FAO Food Balance Sheets [19]. We do not explore the drivers of virtual water trade, such as arable land availability [69–71], or assess how food trade is associated with other environmental issues that could affect future food production, such as climate change. However, our analysis provides novel data on trade patterns in India that can be used in future research to develop policy relevant scenarios to mitigate future food insecurity risks.

### 4.4. Policy implication and future directions

There is substantial interstate trade of cereals in India, but the dominance of rice and wheat as traded crops, and the Northern states as exporting region, potentially increases the vulnerability of India’s food system to changing water availability. Increasing the diversity of crop production could mitigate this risk and simultaneously enhance the diversity of food consumption, which is important for nutritional security. The Indian Central Goods and Service Tax came into effect during 2017, and seeks to streamline the trade of goods and services between states by reducing processing and travel time [72]. This new legislation provides a more accessible market for producers with associated economic benefits, and offers an opportunity to improve the sustainability of the Indian food system through diversification of food supply for consumers [73, 74]. However, it also increases the urgency for interventions to reduce groundwater use and limit food production in over-exploited areas to maintain water security. Recent developments in India such as the Food Smart City initiative could improve the availability of data on food trade and enable states to track risks to their food supply chain [75].

In the context of sustainability research, our study demonstrates the importance of considering trade when quantifying the environmental resource use of food systems. By collating available data on production, consumption and transport, we have explored both the international and sub-national virtual water trade of cereals in India. Our findings are novel for India, where interstate trade is not well understood, and we provide a modelling approach that can be replicated in other settings.

## Data Availability

The data that support the findings of this study are openly available at the following URL/DOI: https://doi.org/10.17037/DATA.00001870.
